# Runx1t1 (Runt-Related Transcription Factor 1; Translocated to, 1) Epigenetically Regulates the Proliferation and Nitric Oxide Production of Microglia

**DOI:** 10.1371/journal.pone.0089326

**Published:** 2014-02-19

**Authors:** Nimmi Baby, Yali Li, Eng-Ang Ling, Jia Lu, S. Thameem Dheen

**Affiliations:** 1 Department of Anatomy, Yong Loo Lin School of Medicine, National University of Singapore, Singapore; 2 Defence Medical and Environmental Research Institute, DSO National Laboratories, Singapore, Singapore; University of Massachusetts Medical, United States of America

## Abstract

**Background:**

Microglia, the resident immune cells of the brain, undergo rapid proliferation and produce several proinflammatory molecules and nitric oxide (NO) when activated in neuropathological conditions. Runx1t1 (Runt-related transcription factor 1, translocated to 1) has been implicated in recruiting histone deacetylases (HDACs) for transcriptional repression, thereby regulating cell proliferation. In the present study, Runx1t1 expression was shown to localize in amoeboid microglial cells of the postnatal rat brain, being hardly detectable in ramified microglia of the adult brain. Moreover, a marked expression of Runx1t1was induced and translocated to nuclei in activated microglia *in vitro* and *in vivo*. In view of these findings, it was hypothesized that Runx1t1 regulates microglial functions during development and in neuropathological conditions.

**Methods and Findings:**

siRNA-mediated knockdown of Runx1t1 significantly decreased the expression level of cell cycle-related gene, cyclin-dependent kinase 4 (Cdk4) and proliferation index in activated BV2 microglia. It was also shown that HDAC inhibitor (HDACi) treatment mimics the effects of Runx1t1 knockdown on microglial proliferation, confirming that microglial proliferation is associated with Runx1t1 expression and HDACs activity. Further, Runx1t1 and HDACs were shown to promote neurotoxic effect of microglia by repressing expression of LAT2, L-aminoacid transporter-2 (cationic amino acid transporter, y+ system), which normally inhibits NO production. This was confirmed by chromatin immunoprecipitation (ChIP) assay, which revealed that Runx1t1 binds to the promoter region of LAT2 and this binding increased upon microglial activation. However, the enhanced binding of Runx1t1 to the LAT2 promoter could not repress the LAT2 expression when the BV2 microglia cells were treated with HDACi, indicating that Runx1t1 requires HDACs to transcriptionally repress the expression of LAT2.

**Conclusion/Interpretation:**

In conclusion, it is suggested that Runx1t1 controls proliferation and the neurotoxic effect of microglia by epigenetically regulating Cdk4 and LAT2 *via* its interaction with HDACs.

## Introduction

Microglia, the resident immune cells of the central nervous system (CNS) transform into an activated state in response to neuropathological conditions, including traumatic brain injury (TBI), causing chronic neurological impairments and neurodegenerative diseases such as the Alzheimer’s disease (AD) [1], [2], [3], [4], [5], [6], [7]. Chronic microglial activation in TBI or AD induces neurotoxicity through excessive release of inflammatory cytokines and cytotoxic molecules such as reactive oxygen and nitrogen intermediates [1], [4], [7], [8], [9]. Further, activated microglia undergo rapid proliferation involving interactions between cell cycle proteins such as cyclins and cyclin-dependent kinases (CDKs) [10], [11], where Cyclin D forms complexes with CDK4 and CDK6 which regulates the G1-S phase transition, a rate-limiting step in the cell cycle progression [11].

Runx1t1 is a member of the RUNX family of transcription factors involved in proliferation and differentiation of haematopoietic stem cells [12], [13]. Runx1t1 mRNA expression has been shown in several human tissues, with its highest expression in the brain and heart [14], [15]. Recent microarray study from our lab showed that Runx1t1 was highly expressed in amoeboid microglial cells when compared to that in ramified microglia [16]. Runx1t1 acts as a transcriptional repressor by recruiting a nuclear co-repressor complex containing HDACs [13], [17], [18], [19], which regulate cell cycle progression by upregulating the cell cycle genes Cdk4, Cdk6 through histone deacetylation [20], [21]. Moreover, HDAC inhibitors (HDACi) such as sodium butyrate, valproic acid and CR2408 have been shown to inhibit cell proliferation by causing cell cycle arrest [20], [21]. It is hypothesised that Runx1t1 may regulate microglial proliferation during development and its activation.

In addition to rapid proliferation, increased production of neurotoxic factors such as nitric oxide (NO) is a characteristic feature of microglial activation. L-aminoacid transporter-2 (LAT2), a member of cationic amino acid transporter system (also known as Y+ system), which has been shown to deplete the availability of arginine to nitric oxide synthase (NOS) enzymes leading to reduction in NO production [22], [23], [24], suggests that LAT2 may have an important role in regulating inflammatory responses. In view of the potential role of LAT2 in inflammation, it was hypothesized that LAT2 is expressed in the microglia and regulates NO production by the activated microglia. Since rapid proliferation and increased production of neurotoxic factors such as NO are characteristic features of microglial activation, the interaction between LAT2 and Runx1t1 in activated microglia was also among the prime focuses of this study.

In this study, we demonstrated the differential expression pattern of Runx1t1 in the normal and activated microglial cells *in vitro* as well as in the TBI and AD rat brain models. Additionally, it has been shown that Runx1t1, in association with HDACs, controls microglia proliferation and epigenetically represses LAT2 gene, which modulates NO production in microglia.

## Materials and Methods

### Ethics Statement

Wistar rats of different age groups (1, 3, 5, 7, 14, 21, 28 d and 3 m) were purchased from the Laboratory Animal Centre, National University of Singapore. All experiments were carried out in accordance with the International Guiding Principles for Animal Research, and approved by the Institutional Animal Care and Use Committee, National University of Singapore (NUS/IACUC/080/10) and DSO National Laboratories Institutional Animal Care and Use Committee (DSOACUC/10/107). All efforts were made to minimize pain and the number of rats used.

### Microarray Analysis

Total RNA was extracted from amoeboid and ramified microglial cells isolated from the corpus callosum of 5 d and 4 w old rat brain sections by LCM. Total RNA was then converted to biotin-labeled cRNA, which was hybridized to the Rat Genome 230 2.0 Array (Affymetrix) [16]. Gene expression was analyzed according to GeneChip Operating Software (GCOS, Affymetrix, CA, USA) [16]. The dataset was submitted to NCBIs Gene Expression Omnibus (http://www.ncbi.nlm.nih.gov/geo/). The dataset can be accessed using the GEO Series accession number GSE29885.

### Normal Postnatal Rats

Wistar rats aged 1, 3, 5, 7, 14, 21, 28 d and 3 m were used for immunofluoresence studies (n = 3, at each age). The rats were anesthetized with xylene-ketamine cocktail (60 mg/kg) and perfused transcardially with Ringer’s solution followed by 2% paraformaldehyde in 0.1 M phosphate buffer. Following perfusion, the brains were removed, post-fixed in the same fixative and cryoprotected in 30% sucrose for 24 h. Frozen sections at 30 µm were cut coronally through the forebrain with a cryostat (Model CM 3050; Leica Instruments GmbH, NUBLOCH, Germany) and mounted onto gelatin-coated slides and stored at −20°C until use.

### Injection of Lipopolysaccharide

In the experimental groups, the rats were given an intraperitoneal (i.p.) injection of 100 µL lipopolysaccharide (LPS) (1 mg/kg; Cat. No. L2654, Sigma-Aldrich, MO, USA). The rats were sacrificed at 3 h after LPS injection; 3 rats receiving an equal volume of saline served as matching controls. For immunostaining, the brains were removed and sectioned, and the tissue sections stored with the same procedure as described above.

### Surgical Preparation for Induction of Fluid Percussion Injury

Rats weighing about 250–300 g body weight were anaesthetized with an i.p injection of Ketamine (75 mg/kg) and Xylazine (10 mg/kg) prior to the surgical procedure. The scalp was shaved and applied with povidine/iodine. The anesthetized animal was mounted and immobilized on a stereotaxic frame with blunt-tip ear bars. An incision was made longitudinally along the dorsal midline of the head using a sterilized scalpel. The scalp and temporal muscle were reflected using sterilized hemostats to expose the bregma and lambda. Using the bregma as a reference point, the guide cannula was lowered so that it was centered on the bregma. A ∼5 mm hole was made at the bregma using a stereotaxic high-speed dental drill and the bone flap was removed. A modified cannula consisting of a head cannula and Female Luer-Lok were fixed firmly with crynaoacrylate glue (dental cement) over the hole until the cannula abuts the dural surface and the Luer-Lok was twisted into place.

### Fluid Percussion Injury (FPI)

The Lateral Fluid Percussion (LFP) device consists of a Plexiglas cylindrical reservoir filled with distilled water. One end of the reservoir has a Plexiglas piston mounted on O-rings and the opposite end has a transducer housed with a 2.6-mm inside diameter male Leur-loc opening. The pendulum was at 90° perpendicular to the ground and just abuts the piston at resting position. The tubing from the percussion device was attached to the head cannula by twisting. The pendulum was released and allowed to fall freely to strike the piston. LFP produced a focal damage in the ipsilateral cerebral cortex and hippocampus by rapidly injecting a small volume of saline epidurally into the closed cranial cavity, producing a brief displacement and deformation of neural tissue. The resulting pressure pulse was measured in atmospheres (ATM) by an extracranial transducer and recorded on a digital storage oscilloscope. The head cannula was then removed from the rat, the burr hole filled with bone wax and the incision closed with surgical staples. Twenty four hours post LFP, the animals were perfused and the brain tissues were collected. Sham-operated animals received similar anaesthesia and surgery, but were not subjected to trauma treatment.

### Alzheimer’s Rat Brain Model

Brain tissues of Samaritan Alzheimer’s Disease rat model fixed with 10% formalin were kindly gifted by Taconic, USA. These tissues were paraffin-embedded on a tissue processor (Model ATP700, Histo-Line laboratories, Italy) and were sectioned on a microtome (Model Leica RM 2165, Germany) at 7 µm thickness and were collected on silane-coated slides for immunofluorescence analysis. According to Taconic**,** Alzheimer’s disease was induced in the rat brain by slow release of ferrous sulfate heptahydrate, L-Buthionine-(S,R)-sulfoximine and Beta-Amyloid (1–42) peptide into the lateral ventricle of the brain.

### Double Immunofluorescence Staining of Rat Brain Tissue

The brain sections were rinsed in phosphate-buffered saline (PBS) and then incubated in 5% normal goat serum diluted in PBS for 1 h at room temperature. Following removal of serum, tissue sections were incubated overnight with a mixture of primary antibodies containing OX-42 (1∶100, mouse monoclonal IgG, Cat. No. CBL1512, Chemicon, USA) and anti-Runx1t1 (1∶100, rabbit polyclonal, Cat.No.sc-28693, Santa Cruz, USA) or anti-LAT2 (1∶100, Cat No. sc-133726, Santa Cruz Biotechnology, USA), at room temperature. On the next day, the sections were washed in PBS and incubated with a mixture of secondary antibodies: FITC-conjugated goat anti-mouse IgG (1∶200, Chemicon, USA) and Cy3-conjugated goat anti-rabbit IgG (1∶200,Chemicon, USA) for 1 h. Subsequently, sections were stained with 4′, 6-diamidino-2-phenylindole (DAPI, 1 µg/mL) for 4 min at room temperature and mounted with a fluorescent mounting medium (DAKO Cytomation, Glostrup, Denmark). Colocalization was examined under a confocal microscope (LSM FV1000; Olympus Company Pte. Ltd,Tokyo, Japan).

### Primary Culture of Microglial Cells

Mixed glial cells were isolated from the cerebrum of 3-days postnatal rats and cultured in a flask containing Dulbecco’s modified Eagle’s medium (Cat. No. 1152, DMEM, Sigma, St. Louis, MO, USA), 10% fetal bovine serum (FBS, HyClone, Logan, UT), 10 ml/L antibiotic-anti-mycotic (Cat. No. A5955, Sigma, USA), 0.1 mM nonessential amino acid (Cat. No. 11140-050, Invitrogen, USA) and 1 ml/L insulin (Cat. No. I-0516, Sigma, USA). The complete medium was replaced at 24 h and then every 3–4 d. Microglial cells were isolated at 10 d with 0.25% trypsin containing 1 mM ethylene diamine tetra acetic acid (EDTA) for 10–20 min at 37°C [25]. With a complete detachment of upper cell layer, microglial cells remained attached to the bottom of the flask. Microglial cells were cultured in a complete medium overnight for their identification. The purity of 90% microglial cells was confirmed by OX-42 labeling.

### BV2 Cell Culture

Murine BV2 microglial cell line [26], [27], [28] cultured in 75 cm^2^ culture flasks containing DMEM supplemented with 10% FBS and 1% antibiotic antimycotic solution, at 37°C in a humidified atmosphere of 5% CO_2_ and 95% air incubator. Cells were plated on 96-well plates at about a density of 1.0×10^6^ per 100 mm^2^ for RNA isolation, at 2.0×10^5^ per well on a 24-well plate for immunocytochemistry. On the following day, cells were plated on poly-L-lysine coated coverslips in 24 well plates and incubated with either LPS (1 µg/mL) for 3 h or HDAC inhibitor (5 mM sodium butyrate) overnight [29]. Some plates of cells were pre-treated with HDAC inhibitor overnight before LPS treatment. The complete medium was replaced by basic medium while cells are treated with LPS. The cells cultured in the basic medium served as the control. The time point (3 h) and dosage (1 µg/ml) of LPS treatment have been chosen based on results obtained from MTS assay and previous reports [27], [30].

### Cytotoxicity Assay (MTS Assay)

Cytotoxicity assay was performed using MTS assay kit (Cat No. G3582) which contains 3-[4,5-dimethylthiazol-2-yl]-5-(3-carboxymethoxyphenyl)-2-(4-sulfophenyl)-2H tetrazolium, inner salt, MTS and an electron coupling reagent (phenazine ethosulfate; PES). BV2 cells were seeded in a 96-well plate (5000 cells/well) containing 100 µl culture medium and treated with different concentrations of LPS (0.1 µg/ml–10 µg/ml) at 37°C for 1–6 h. MTS reagent (20 µl/well) was added to the culture and incubated at 37°C for 1–4 h. The absorbance was recorded at 490 nm using a 96-well plate reader (UV max, Molecular Devices, USA).

### Knockdown of Runx1t1 in BV2 Microglial Cells by siRNA

Expression of Runx1t1 in BV2 microglial cells was inhibited using specific siRNA (Silencer siRNA transfection, Cat No. 4390771, Ambion, Inc. USA). Pre-designed Runx1t1 siRNA and Scrambled siRNA were obtained from Ambion (Table 1). The siRNA transfection in microglial cells was performed according to the manufacturer’s instruction. BV2 microglial cells were resuspended in OPTIMEM medium at a concentration of 1×10^5^ cells/ml. Transfection complexes were prepared by mixing RNAiMAX Lipofectamine transfection agent (Cat No. 13778-150, Ambion) and siRNA (20 nM) in OPTIMEM Serum-free Medium (Invitrogen Life Technology, USA ). Microglial cells were then mixed with transfection complexes and incubated for 24 h in 6-well (2×10^5^ cells/well) or 24-well (4×10^4^ cells/well) plates.

**Table 1:** Primer sequences used for RT–PCR.

Genes	Sense	Antisense	Size (bp)
Runx1t1	ACGAACAGCTGCTTCTGGAT	TGCTTGGATGTTCTGAGTGC	153
Cdk4	CAGGCCGCTTAGAAACTGAC	CAATGTTGTACGGCTGATGG	178
m βactin	GGATTCCATACCCAAGAAGGA	GAAGAGCTATGAGCTGCCTGA	103
R βactin	CCTAGAAGCATTTGCGGTGCAGGATG	TCATGAAGTGACGTTGACATCCGT	246
mLAT2 Primer (ChIP)	GGAGTCGCTGGTACAGTTCTATTT	AAAAGTGACAGCAAATGAGAGTCA	123
mGAPDH primer (ChIP)	CCTCTGCGCCCTTGAGCTAGGA	CACAAGAAGATGCGGCCGTCTC	158
Runx1t1 siRNAConstruct 1 (s221449)	GCAUCUCGAUCAUCUGUUA	UAACAGAUGAUCGAGAUGC	
Runx1t1 siRNAConstruct 2 (s221450)	GGUGAACUCUACUUUGACA	UGUCAAAGUAGAGUUCACC	

Runx1t1 protein expression in microglial cells was analyzed by immunofluorescence (n = 3). Transfected BV2 microglial cells were fixed with 4% paraformaldehyde in phosphate buffer for 30 min at room temperature. Following incubation in normal goat serum, all coverslips with adherent cells were incubated in rabbit anti-Runx1t1 (1∶100, Cat.No.sc-28693, Santa Cruz, USA) at 4°C overnight. Subsequently, the cells were washed with PBS and incubated with FITC-conjugated lectin and Cy3-conjugated anti-rabbit IgG (1∶200, Sigma) for 1 h. Cells were finally counterstained with DAPI (1 µg/ml, Invitrogen, USA).

### BrdU Incorporation Assay in BV2 Microglial Cells

Microglial cells were transferred onto poly-L-lysine-coated coverslips in 24-well plates. The cells subjected to different treatments (Runx1t1 siRNA and HDACi, n = 3) were incubated with BrdU (10 µmol/l) for 2 h and fixed with 4% PF for 20 min at room temperature. Subsequently cells were treated with 2N HCl at 37°C for 30 min, blocked with 2% normal goat serum for 30 min, and incubated with anti-BrdU monoclonal antibody (1∶1000, Cat No. B-2531, Sigma,USA) overnight at 4°C. Further, the cells were incubated with goat anti-mouse secondary antibody conjugated with Cy3 (1∶200, Sigma) for 1 h at room temperature and counter-stained with lectin (1∶100) for scoring the total microglial cell number. The percentage of BrdU positive cells was determined by randomly scoring 5 fields for each experimental and control groups in each independent experiment (n = 3). The proliferation index is expressed as mean ± SD of the percentage of BrdU positive cells.

### RNA Extraction and Real Time Reverse-transcription Polymerase Chain Reactions (RT-PCR)

The total RNA was extracted from primary microglial cells and BV2 microglial cells subjected to various treatment (LPS, HDACi, scrambled and Runx1t1 siRNA transfection) using RNeasy Mini kit (Qiagen, Valencia, CA, USA). The concentration of RNA was quantified with a Sphectrophotometer (Nanodrop; Model No. ND1000, Thermo Scientific, MA, USA). Reverse transcription was carried out as follows: A mixture of 2 µg total RNA and 2 µl Oligo (dT) was heated at 70°C for 5 min, rapidly cooled down on ice and mixed with reaction mixture containing 5 µl M-MLV RT 5X Buffer (Cat. No M531A, Promega, USA), 0.5 µl deoxyribonucleotide triphosphate (dNTP, 25 µM, Cat No. U1240, Promega, USA), 0.7 µl RNAse inhibitor (2500U, RNasin Cat. No N211A, Promega, USA) and 1 µl M-MLV reverse transcriptase (10000 u; Cat. No M170A, Promega, USA). The reaction was made up to 25 µl with RNA-free water and then incubated at 42°C for 1 h and at 70°C for 10 min. cDNA was synthesized and stored at −80°C until its use. RT-PCR was carried out on an Applied Biosystems 7900HT thermal cycler instrument using QuantiTect SYBR Green PCR Kit (Cat. No.204141, QIAGEN, USA) according to manufacturer’s instructions. For RT-PCR, reaction mixture containing 5 µl of QuantiTect SYBR Green PCR Master Mix, 1 µl of 10 µM forward primer, 1 µl of 10 µM reverse primer (Table 1), cDNA template and RNAse-free water was prepared and transferred into 96 well plates. The PCR was performed under the following conditions: an initial denaturation at 95°C for 15 min, followed by 45 cycles of denaturation at 94°C for 15 s, and annealing at 60°C for 25 s, with a final extension at 72°C for 12 s. Expression level of target genes was measured in triplicate and was normalized to βactin, an internal control. The RT-PCR products were loaded to 2% agarose gel electrophoresis, stained with 1 mg/mL ethidium bromide, and photographed using ChemiGenius2 image analyzer (SYNGENE, Cambridge, UK). The predicted size and single band confirmed the amplicons and that the primers used were specific. Fold change of target genes’ expression was calculated according to the formula: fold change = 2^−[[Ct (control) gene X-Ct (control) actin][Ct (activated) gene X-Ct (activated) actin]]^ [31].

### Immunofluorescence Staining of Microglial Cells

Primary microglial cells or BV2 microglial cells were plated onto poly-L-lysine coated cover slips in a 24-well plate. For immunofluorescence, the cells subjected to different treatments were fixed with 4% paraformaldehyde in phosphate buffer for 30 min at room temperature (n = 3). Following incubation in normal goat serum, all coverslips with adherent cells were incubated in a mixture of antibodies containing OX-42 (1∶200, Chemicon, USA) with anti-Runx1t1 (1∶100, Cat.No.sc-28693, Santa Cruz, USA), or anti-LAT2 (1∶100, Cat No. sc-133726, Santa Cruz Biotechnology, USA), or anti-H3acetylated(Lys9/14) (1∶100, Cat No. 17–615, Millipore, USA) at 4°C overnight. The coverslips were washed and incubated with FITC-conjugated goat anti-mouse IgG (1∶100, Chemicon, USA), or Cy3-conjugated goat anti-rabbit IgG (1∶100, Chemicon, USA) for 1 h at room temperature. Cell nuclei were counterstained with DAPI (1 µg/mL) for 2 min. The coverslips were then washed and mounted with a fluorescent mounting medium (DAKO Cytomation, Glostrup, Denmark). All images were captured under a confocal microscope (LSM FV1000; Olympus Company Pte. Ltd, Tokyo, Japan).

### Western Blot Assay

Total protein of BV2 cells exposed to different treatments was extracted separately using the Protein Extraction Kit (Prod#78501, Thermo Fish Scientific Inc., Rockford, IL, USA), Halt™ Protease Inhibitor Cocktail Kit (Prod#78410, Thermo Fish Scientific Inc., Rockford, IL, USA) and nuclear protein extraction kit (Cat No.2900, Millipore,USA). The protein level was quantified using a protein assay kit (Cat. No.500-0007, Bio-Rad, Hercules, CA, USA). Twenty µg of protein extracts were separated on 10% SDS-polyacrylamide gels and transferred to polyvinylidene difluoride transfer membranes. The membranes were blocked with 5% non-fat dry milk and incubated with primary antibodies over night at 4°C. The primary antibodies used are as follows: rabbit anti-Runx1t1, (1∶500, Cat.No.sc-28693,Santa Cruz, USA), rabbit anti-LAT2 (1∶500, Cat No. sc-133726, Santa Cruz Biotechnology, USA), rabbit anti-cdk4 (1∶500,Cat.No sc-260 Santa Cruz, USA) and mouse anti-β-actin monoclonal antibody (Cat No: A1978, Sigma-Aldrich, St. Louis, MO, USA). The next day, the membranes were incubated with the horseradish peroxidase-conjugated secondary antibody (Cat. No.7074, Cell Signalling Technology, USA) for 1 h. The immunoproducts were detected using a chemiluminescence detection system according to the manufacturer’s instructions (Supersignal West Pico Horseradish Peroxidase Detection Kit, Pierce Biotechnology, IL, USA) and developed on a film. The optical density of each protein band was quantified by a scanning densitometer and Quantity One Software, version 4.4.1 (Bio-Rad, CA, USA). Each lane of protein band density was normalized with corresponding β-actin density.

### Nitric Oxide Assay (NO Assay)

Culture medium was collected from the culture of BV2 cells transfected with scrambled siRNA or Runx1t1 siRNA and HDACi-treated BV2 cells. The nitric oxide production in the culture medium was quantified colorimetrically using nitric oxide assay kit with a Griess reagent system (US Biological, USA; Cat. No.2577-01). Equal volumes of culture medium and the Griess reagent (0.1%N-(1-naphthyl) ethylenediamine dihydrochloride, 1% sulfanilamide, and 2.5% H3PO4) were mixed and incubated for 10 min at room temperature. The absorbance at 540 nm was determined with an E MAX precise microplate reader (Molecular Devices, USA).

### Chromatin Immunoprecipitation (ChIP) Assay

Chromatin immunoprecipitation was performed using Chromatin Immunoprecipitation Kit (EZ-ChIP, Cat No: 17–371, Millipore, Inc., USA). BV2 microglial cells were cross-linked with 1% formaldehyde for 10 min at room temperature and quenched with 0.125M glycine for 5 mins at room temperature. Then the cells were washed with ice-cold PBS containing protease inhibitors for two times, harvested and sonicated using SDS lysis buffer (50 mM Tris, pH8.1, 10 mM EDTA, 1% SDS, Cat. No. 20–163, Millipore, USA). The chromatin shearing was carried out using a sonicator (Cat. No. UCD-200, Bioruptor, Diagenode Inc., USA) on high power for 3×10 cycles (30 s ON/30 s OFF) and the chromatin extracts were precleared with 50% suspension of CHIP blocked protein G agarose (Cat. No. 16–201D, Millipore, USA) for 1 h and immunoprecipitated with 8 µg of anti-Runx1t1 antibody (Cat.No.sc-28693, Santa Cruz, USA), or anti-polymerase II antibody (Cat. No.05-623B,Millipore, Inc., USA) or IgG control antibody (Cat. No.sc-2027, Santa Cruz, USA) overnight, respectively at 4°C. Immune complexes were recovered followed up by consecutive washes with low salt buffer, high salt buffer, LiCl immunecomplex buffer, and Tris–EDTA buffer (Cat. No.20–157, Millipore, Inc., USA). The immunocomplex was extracted in elution buffer and the protein–DNA crosslink was reverted by overnight treatment at 65°C. The samples were subjected to RNase A digestion (10 µg; 37 min at 37°C) and proteinase K digestion (10 µg; 2 h at 37°C). The immunoprecipitated DNA was purified with PCR purification kit (Cat. No. 28104, QIAGEN, USA). Input DNA was purified from the precleared chromatin. Quantitative PCR was performed using the Power SYBR Green PCR master mix on an Applied Biosystems 7900HT thermal cycler with 2 µl of immunoprecipitated DNAs and input DNAs using primers against LAT2 promoter region (Table 1) and GAPDH promoter region (Table 1).

### Statistical Analysis

Data used for statistical analysis were derived from three to four independent experiments and presented as means +/−SD. Statistical significance was evaluated by either the Student’s t-test or one-way ANOVA. Results were considered as significant at *p*<0.05 or *p*<0.01.

## Results

### Runx1t1 Immunoexpression in Microglial Cells Varies with Age in Normal Rats

**Figure 1:**
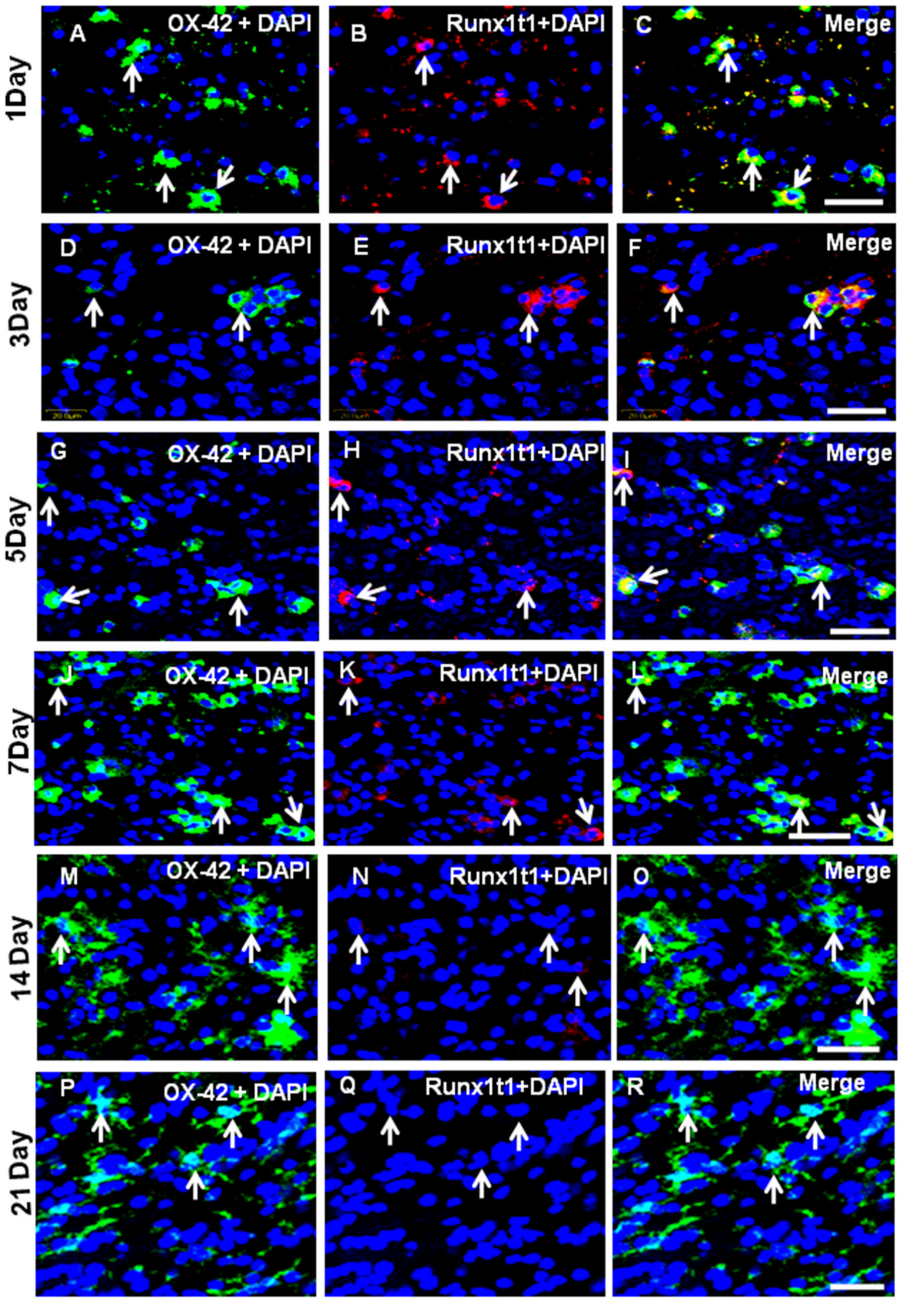
Co-localisation of Runx1t1 and OX42 in the postnatal rat brain by double immunofluorescent staining. Confocal images showing the expression of Runx1t1 (B,E,H,K,N,Q; red) in OX42-positive (A,D,G,J,M,P; green) microglia (C,F,I,L,O,R; yellow) from the corpus callosum of 1, 3, 5,7, 14& 21 days rat brains, respectively. Note that the expression of Runx1t1 (arrows) decreases with age. Nuclei are stained with DAPI. Scale bar: 20 µm.

**Figure 2:**
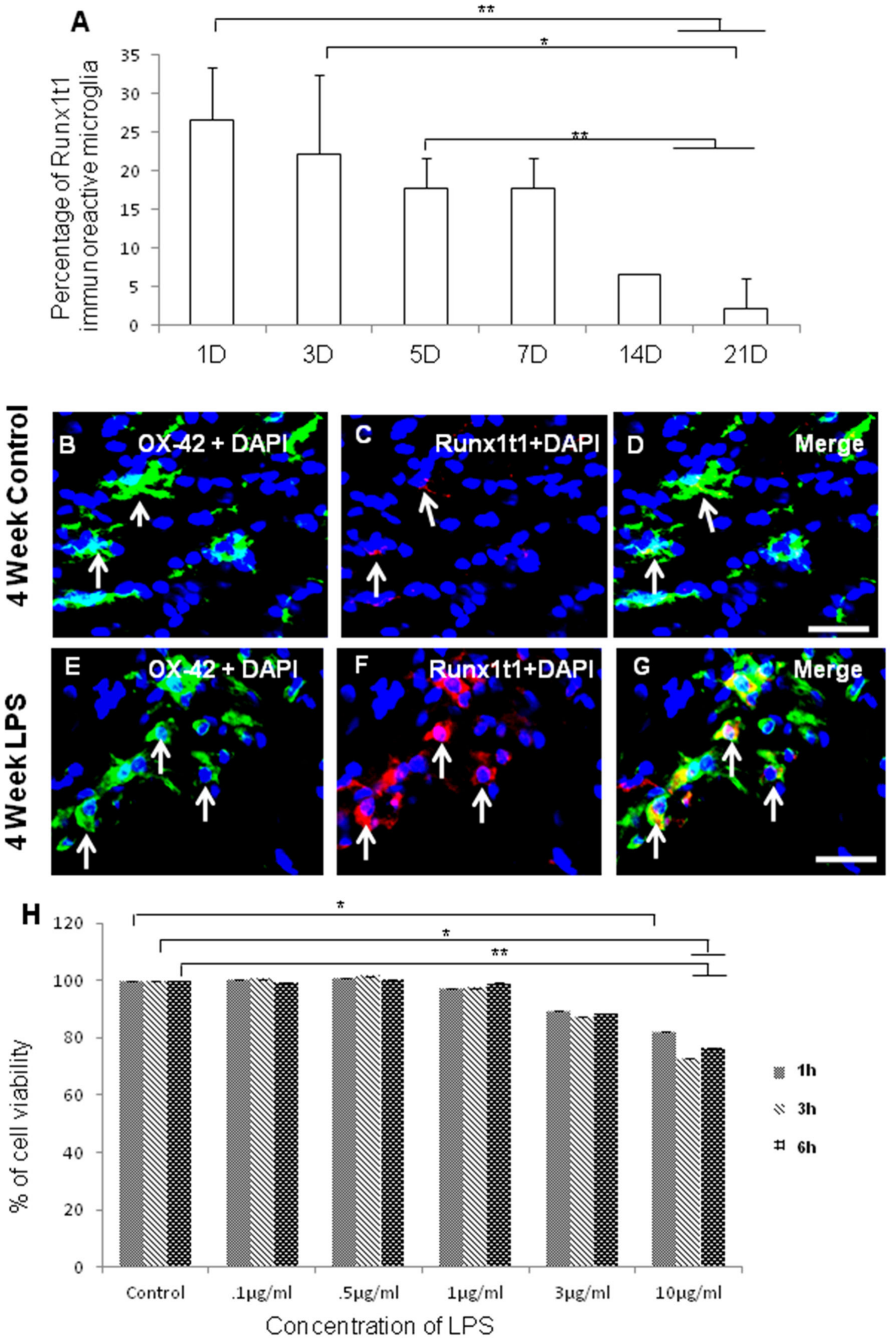
Quantitative analysis showing the percentage of Runx1t1 immunoreactive microglia in rat brain. **A.** Quantitative analysis shows that number of Runx1t1 immunoreactive microglia decreases with age in normal rats. Significant differences between groups (1D *vs*14D, 1D *vs* 21D, 3D *vs* 21D, 5D *vs* 14D, 5D *vs* 21D) are indicated by *p<0.05, **p<0.01 (n = 3). **B–G.** Confocal images showing the colocalization of Runx1t1 (C,F; red) and OX42 (B,E; Green) in control (B–D) and LPS-treated (E–G) activated microglia from the corpus callosum of 4 week rat brain. Nuclear translocation of Runx1t1 is evident in LPS-treated activated microglia (F;arrows). Nuclei are stained with DAPI. Scale bars: 20 µm. **H.** MTS assay shows the effect of various concentrations of LPS (0.1 µg/ml–10 µg/ml) on the viability of BV2 microglial cells. Treatment of these cells with different concentrations of LPS (0.1 µg/ml–10 µg/ml) for 1 h, 3 h and 6 h revealed that LPS did not affect the cell viability up to 1 µg/ml concentration. Cell viability was decreased with further increase in the concentration. Significant differences between groups (Control *vs* LPS (3 µg/ml), Control *vs* LPS (10 µg/ml) are indicated by *p<0.05, **p<0.01 (n = 3).

Immunofluorescence analysis by confocal microscopy showed that Runx1t1 was expressed in OX42-positive amoeboid microglial cells (AMC) found in the corpus callosum of 1–5 d old rat pups (Fig. 1 A–I). The intensity of Runx1t1 expression in microglia appears to decline gradually from 7 d onwards (Fig. 1 J–L). In rats aged 14 d and 21 d, Runx1t1 expression was almost undetectable in the microglia which appeared to be ramified (Fig. 1M–R). Quantitative analysis showed that the number of Runx1t1 immunoreactive microglia decreased with age in normal rats (Fig. 2 A).

### Runx1t1 Expression is Upregulated in Activated Microglia Both in vivo and in vitro

As microglial cells are known to respond to neuroinflammation and injury, we have examined if there is any change in expression of Runx1t1 in microglia exposed to LPS, which is an endotoxin causing inflammation in the brain. Runx1t1 immunoreactivity was found to be induced in activated microglia distributed in the corpus callosum of 4 weeks old rats, 3 h after injection of LPS (Fig. 2E–G) when compared to the control (Fig. 2B–D). Nuclear translocation of Runx1t1 protein was also evident in some activated microglia of LPS treated groups (Fig. 2F).

**Figure 3:**
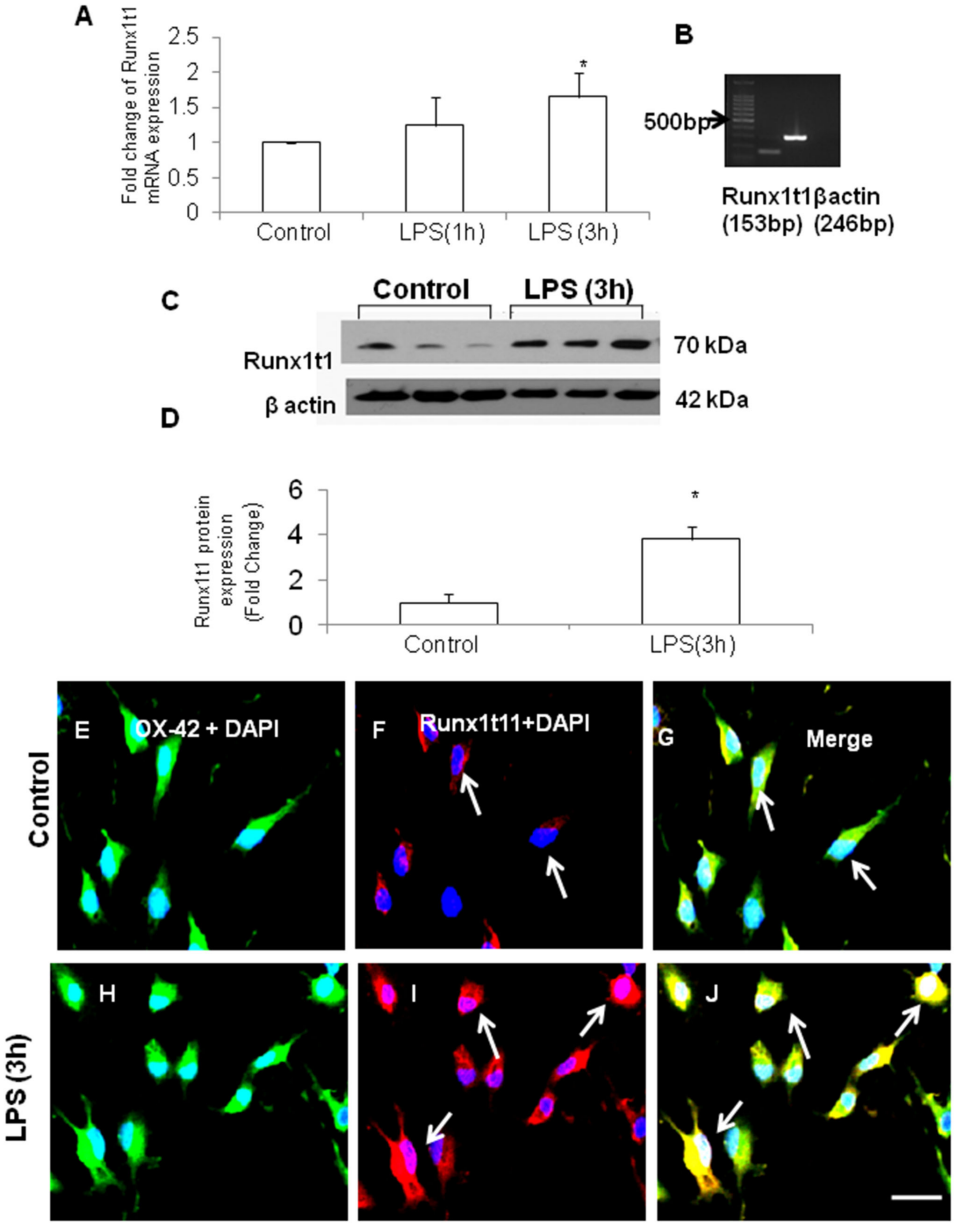
Expression of Runx1t1 in LPS-activated primary microglia. **A–B.** qRT PCR shows that Runx1t1 mRNA expression is markedly upregualated in LPS-treated primary microglia. The data are normalized with the β-actin (B) and presented as mean ± S.E. Control vs LPS. **C–D.** Western blot analysis shows that there is a significant upregulation of Runx1t1 protein expression in LPS-treated primary microglial cells when compared to control. Runx1t1 (70 kDa) and β-actin (42 kDa) immunoreactive bands are shown (C). Significant differences between the groups are indicated by *p<0.05 (n = 3). **E–M.** Confocal images showing the co-expression of Runx1t1 (F,I; Red) and OX42 (E,H; Green) in primary culture microglia (E–J) treated with LPS for 3 h (H–J). Runx1t1 expression appears to be markedly increased after 3 h of LPS treatment (I; arrows). Nuclear localization of Runx1t1 is evident in activated microglia (I; arrows). Nuclei are stained with DAPI. Scale bars: 50 µm.

MTS assay was performed to determine the toxicity of LPS and the viability of BV2 microglial cells after treatment with various concentrations of LPS (0.1 µg/ml–10 µg/ml) for 1 h, 3 h and 6 h. The viability of cells treated with up to 1 µg/ml concentration of LPS was near normal levels compared to untreated control cells (Fig. 2H). However, cell viability seemed to reduce at 3 µg/ml and 10 µg/ml concentrations of LPS. Further, mRNA and protein expression levels of Runx1t1 were detected in primary microglial cells *in vitro* and were significantly upregulated in cells exposed to LPS for 3 h (Fig. 3A–M). Based on these findings, the time point (3 h) and dosage (1 µg/ml) of LPS treatment have been used for all further studies.

Immunofluorescence analysis further confirmed that Runx1t1 immunoreactivity was increased in primary culture microglial cells treated with LPS for 3 h (Fig. 3 H–J) when compared to control (Fig. 3 E–G). Nuclear translocation of Runx1t1 protein was evident in activated microglial cells (arrows in Fig. 3I).

**Figure 4:**
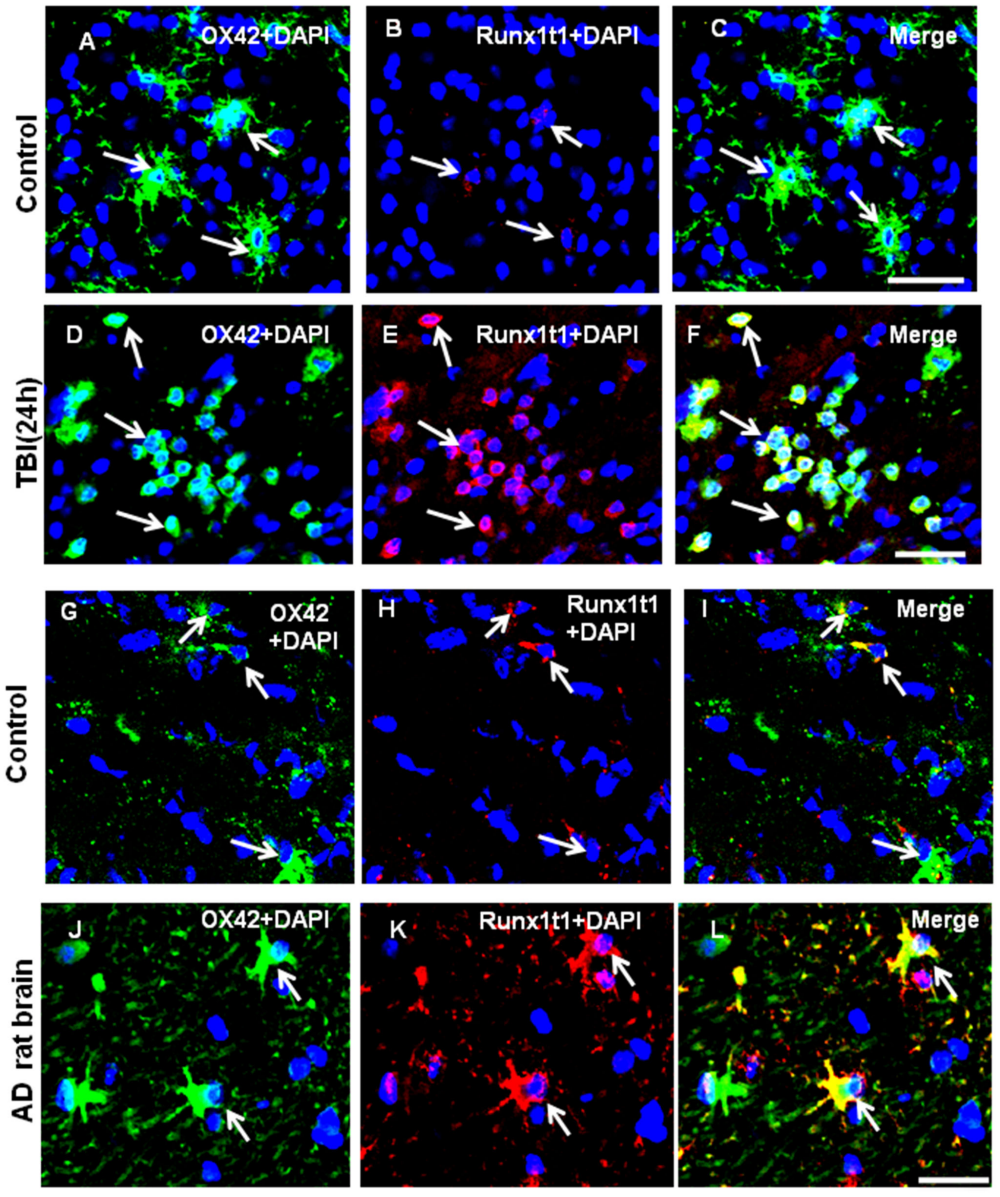
Runx1t1 protein expression in the activated microglial cells of TBI and AD rat brain models. **A–L.** Confocal images showing the colocalization of Runx1t1 (Red) and OX42 (Green) in activated microglia in the CA1 region of hippocampus from TBI (A–F) or AD (G–L) rat models. Control sections were obtained from the sham-operated rat brains (A–C). Runx1t1 expression appears to be increased in activated microglial cells 24 h after TBI (D–F; arrows) when compared with control (A–C). **G–L.** Runx1t1 expression appears to be increased in activated microglial cells in the hippocampus of Alzheimer’s rat model (J–L) when compared to control sections (G–I). Nuclei are stained with DAPI. Scale bars: 50 µm.

Since microglia is activated in response to brain injury and neurodegeneration, we have analyzed Runx1t1 expression in microglia of the brain from TBI and AD rat models. After TBI which is focal and specific to the CA1 region of hippocampus, the activated microglia appeared amoeboid in shape in the CA1 region of hippocampus and exhibited induced-expression of Runx1t1 protein (Fig. 4D–F) when compared with that of the sham operated control animals (Fig. 4A–C). We have also analysed the expression of Runx1t1 in microglia of the brain from rat AD model. In age-matched control rat brains, Runx1t1 expression was hardly detectable (Fig. 4G–I), whereas in the brain of AD rat model, Runx1t1 expression was found to be induced in activated microglia (Fig. 4J–L).

### siRNA-mediated Knockdown of Runx1t1 Suppresses Microglial Proliferation and Expression of Cell-cycle Related Genes in BV2 Microglia

**Figure 5:**
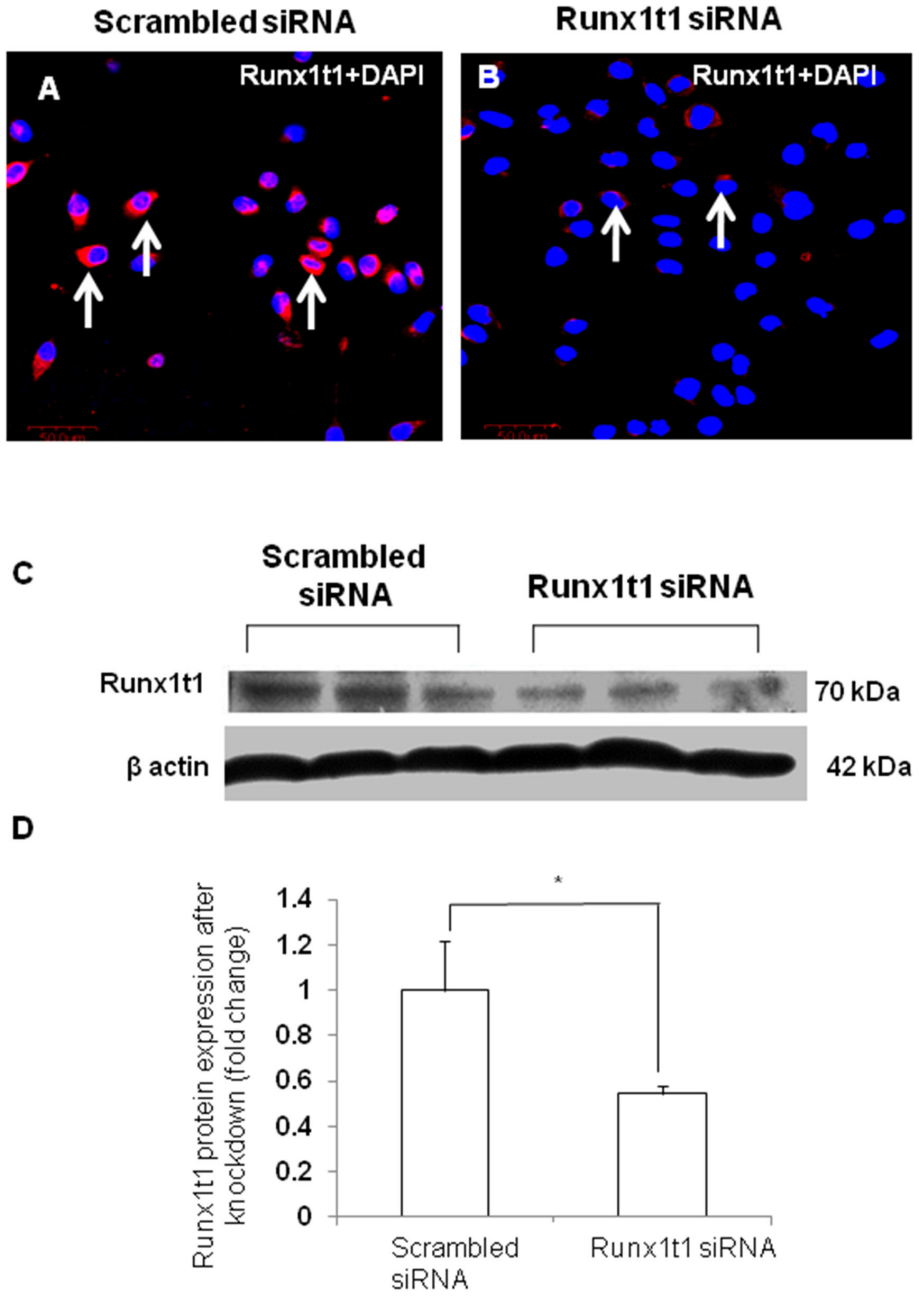
Transfection efficiency of Runx1t1 siRNA in BV2 microglial cells. **A–B.** Confocal images showing the Runx1t1 expression in BV2 microglial cells transfected with scrambled siRNA (A) and Runx1t1 siRNA (B). Runx1t1 expression is hardly detectable in microglia 24 h afterRunx1t1 siRNA transfection (B). Nuclei are stained with DAPI. **C–D.** Western blot analysis showing a significant downregulation of Runx1t1 protein in Runx1t1 knockdown BV2 microglial cells when compared to that transfected with scrambled siRNA. Runx1t1 (70 kDa) and β-actin (42 kDa) immunoreactive bands are shown (C). Significant differences between the two groups are indicated by *p<0.05(n = 3). Scale bars: 50 µm.

**Figure 6:**
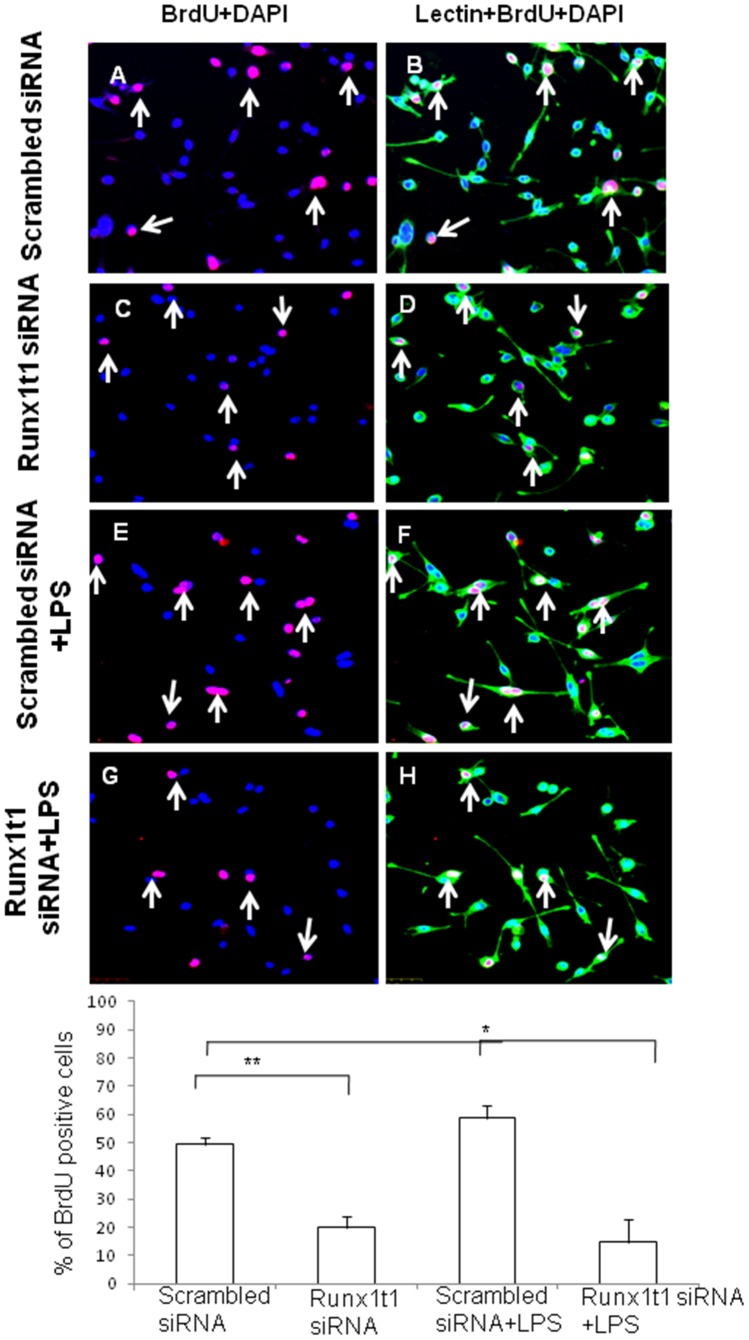
Confocal images showing the BrdUimmunostaining in lectin-positive BV2 microglia (A,C,E,G; red). The number of BrdUpositive microglial cells (C,G: arrows) appears to be decreased at 24 h after Runx1t1 knockdown when compared to the cells transfected with scrambled siRNA. Nuclei are stained with DAPI. Scale bars: 50 µm. **I.** Quantitative analysis shows a significant decrease in number of BrdUimmunoreactive cells in activated microglia cultures transfected with Runx1t1siRNA for 24 h. Significant differences between groups (scrambled siRNA *vs* Runx1t1 siRNA, scrambled siRNA +LPS *vs* Runx1t1 siRNA+LPS) are indicated by *p<0.05, **p<0.01 (n = 3).

**Figure 7:**
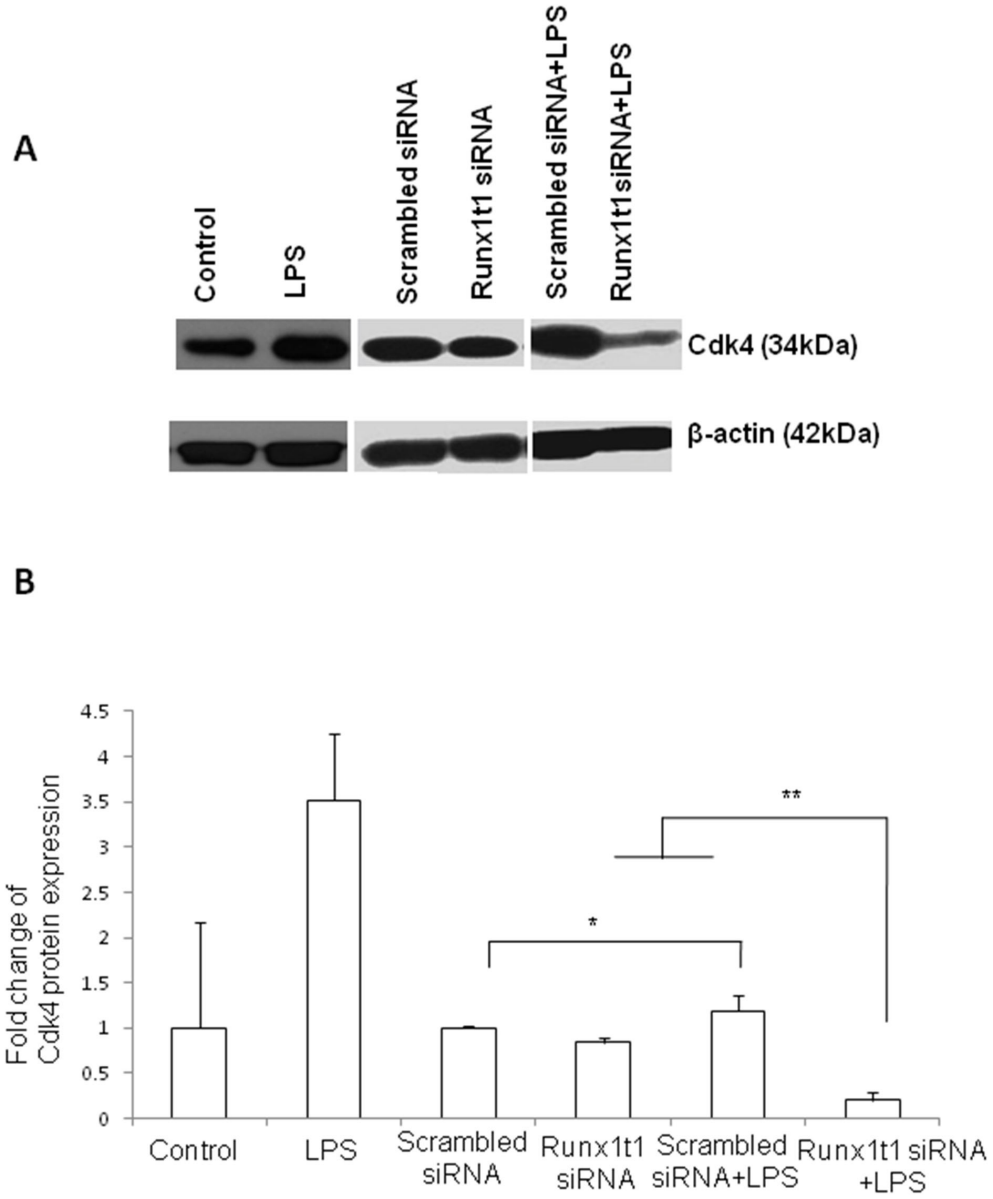
Cdk4 protein expression in LPS-activated Runx1t1 knockdown BV2 microglia. **A–B.** Western blot analysis shows that Cdk4 protein expression is upregulated in LPS-treated BV2 microglial cells (A–B). However, a significant downregulation of Cdk4 protein expression is detected in LPS-treated BV2 microglia after knockdown of Runx1t1 (A–B). The significant difference between the groups (scrambled siRNA *vs* scrambled siRNA +LPS, Runx1t1 siRNA *vs* Runx1t1 siRNA+LPS and scrambled siRNA +LPS *vs* Runx1t1 siRNA+LPS) are indicated by *p<0.05, **p<0.01(n = 3).

In order to study the role of Runx1t1 in microglia, BV2 microglial cells were transfected with siRNA which was designed to knockdown the expression of endogenous Runx1t1. The transfection was considered to be efficient since the Runx1t1 mRNA expression level was found to decrease by 90% in transfected cells (data not shown). Further, there was no marked change in cell viability after transfection of Runx1t1 siRNA and CY3-conjugated GAPDH siRNA, the positive control (data not shown). Immunofluorescence analysis showed that Runx1t1 protein expression decreased considerably and was hardly detectable in BV2 microglial cells after siRNA knockdown of Runx1t1, when compared to that in cells transfected with scrambled siRNA (Fig. 5 A, B). This was further confirmed by western blot analysis which showed a significant reduction in Runx1t1 protein after the knockdown of Runx1t1 with siRNA (Fig. 5 C, D). In addition, BrdU incorporation assay revealed that the knockdown of Runx1t1 significantly decreased the mitotic index of BV-2 microglia cells (Fig. 6 A–H, I), indicating the role of Runx1t1 on microglial proliferation. This was further confirmed by a significant downregulation of the expression of cell cycle-related protein, Cdk4 in BV2 microglial cells transfected with Runx1t1 siRNA (Fig. 7 A–B).

### HDACi Inhibits Microglial Proliferation, Mimicking the Effect of Runx1t1 Knockdown in Microglia

**Figure 8:**
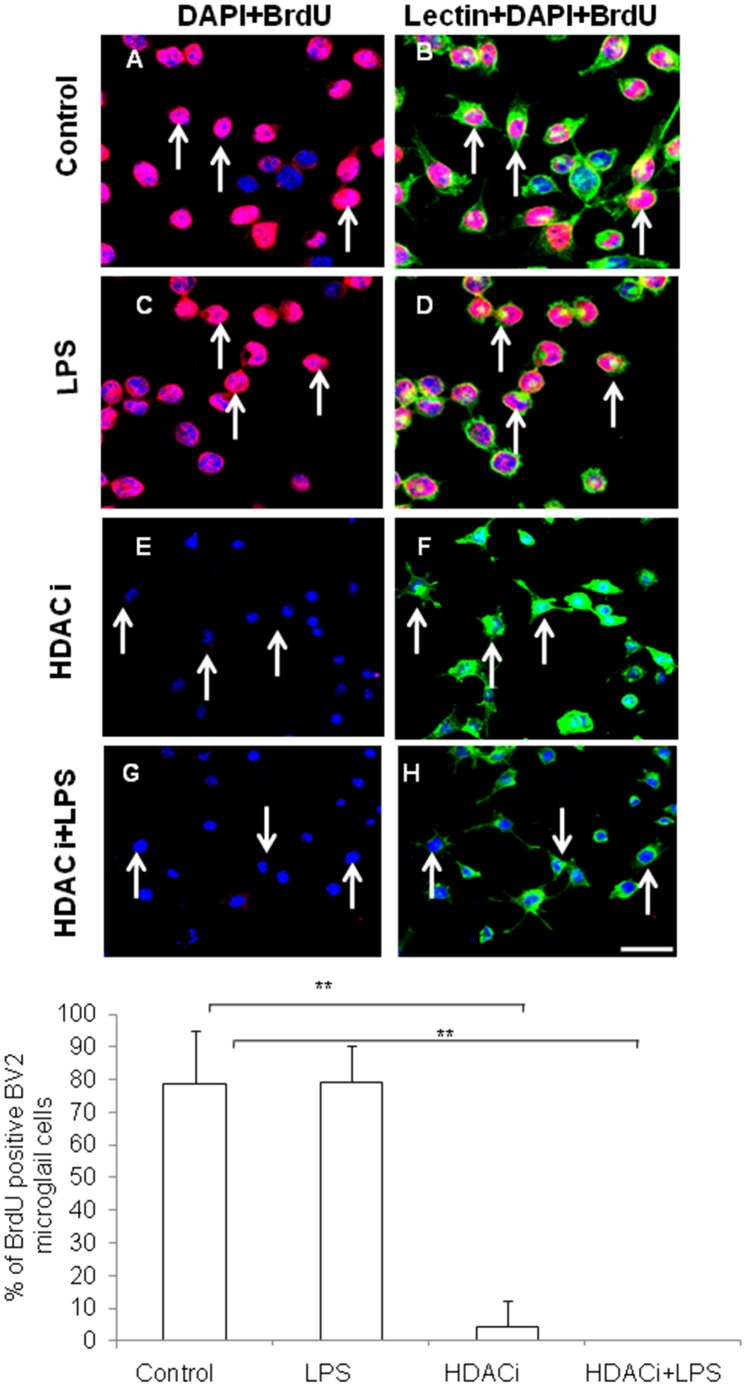
Confocal images showing inhibition of BrdU incorporation in BV-2 microglial cells treated with HDAC inhibitor. **A–H.** Microglia cells are immunostained with BrdU [(A,C,E,G); red] and counterstained with lectin and DAPI. The number of BrdU positive cells (E,G);arrows) decreased at 24 h after HDAC inhibitor treatment. Scale bars: 50 µm. **I.** Quantitative analysis shows a significant decrease in number of BrdUimmunoreactive cells in BV2 microglial cells treated with HDAC inhibitor for 24 h. Significant differences between groups (control *vs* HDACi, Control *vs* HDACi+LPS) are indicated by **p<0.01 (n = 3).

Treatment with HDACi (sodium butyrate, 5 mM) showed a similar effect to Runx1t1 knockdown on microglial proliferation. BrdU incorporation assay showed a significant decrease in number of BrdU**^+^**microglial cells following treatment of HDACi with or without LPS in comparison to that of control as well as LPS-treated cells (Fig. 8 A–H, I).

### Runx1t1 Knockdown and HDACi Treatment Induces Histone Acetylation in Microglia

**Figure 9:**
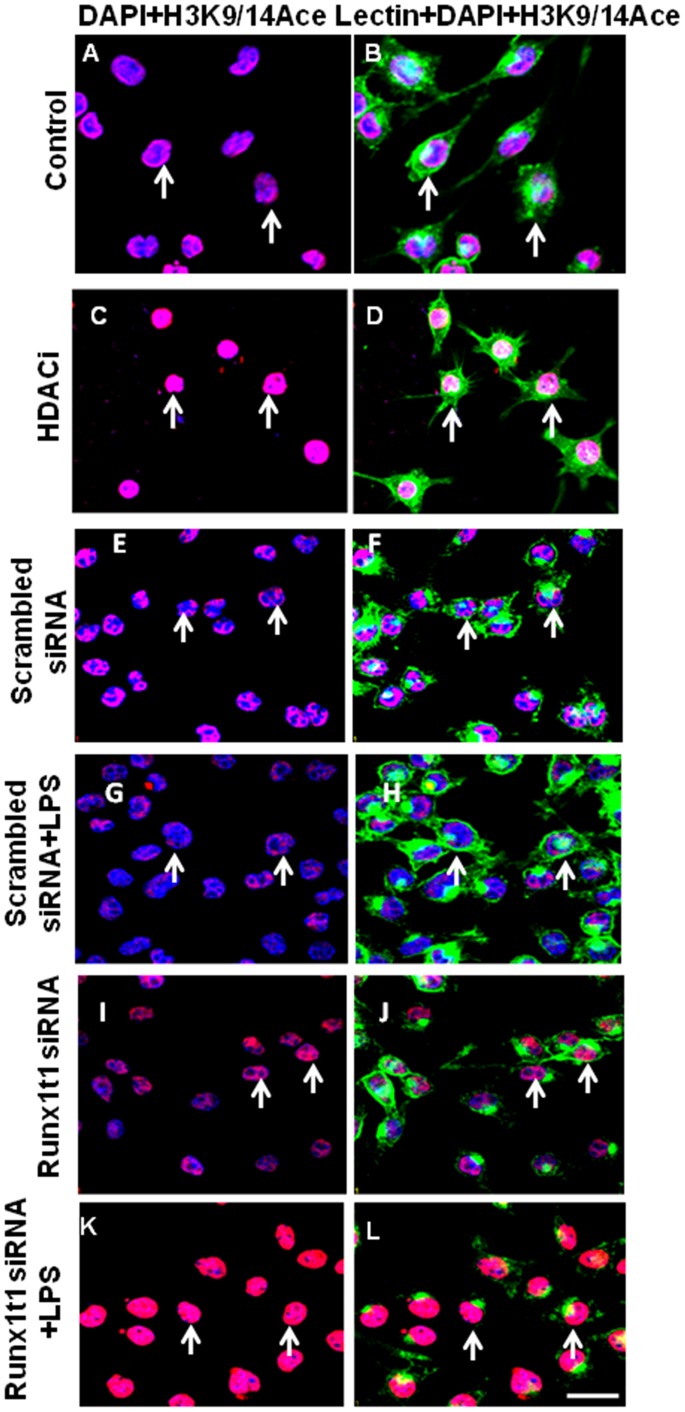
Confocal images showing acetyl-histone H3 (K9/14) immunostaining (Red) in BV2 microglial cells which are counterstained with lectin (Green) and DAPI (Blue). HDACi treatment increased acetyl-histone H3 staining in microglial cells(C,D) when compared to control cells (A,B). Further, knockdown of Runx1t1 markedly increased acetyl-histone H3in microglial cells treated with or without LPS (I–L) when compared to scrambled siRNA transfected cells (E–H). Scale bars: 50 µm.

Since HDACi treatment has been reported to increase the histone acetylation [32], [33], the acetylation status of BV-2 microglia (both control and activated) was examined after HDACi treatment and Runx1t1 knockdown using anti-acetyl-histone H3 (K9/14) antibody (Fig. 9). A marked increase in acetylation was observed in the nuclei of microglia after HDACi treatment (Fig. 9 C–D) and Runx1t1 siRNA knockdown (Fig. 9 I–J, K–L) as revealed by immunofluorescence analysis.

### HDACi Treatment Downregulates the Expression Level of Cdk4 as in Runx1t1 Knockdown Microglial Cells

**Figure 10:**
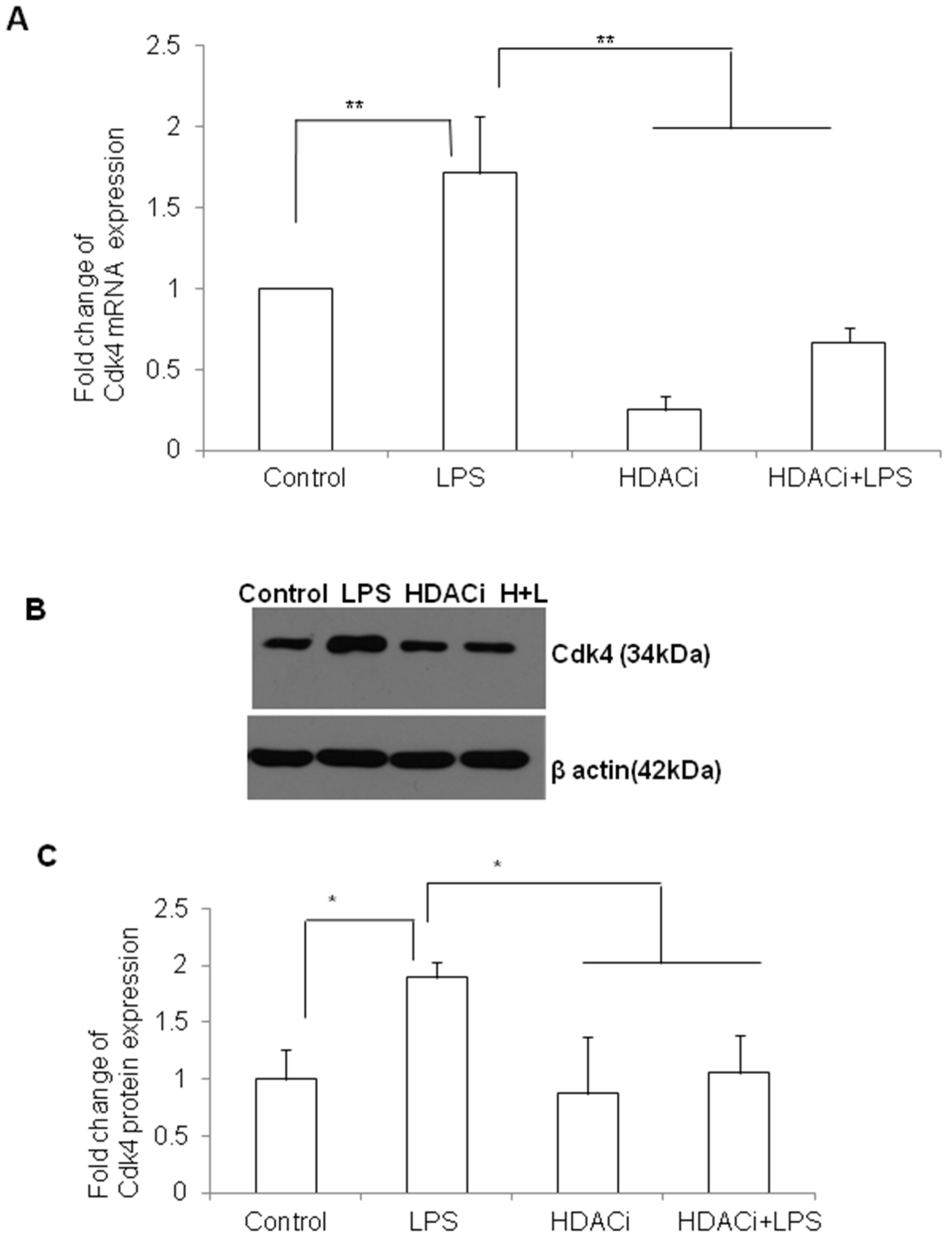
Cdk4 mRNA and protein expression in BV2 microglia treated with HDACi. **A.** qRT-PCR analysis shows that Cdk4 mRNA expression is significantly upregulated in LPS-treated BV2 microglia and however, downregulated when cells are treated with HDACi. **B–C.** Western blot analysis also showed a similar trend ofCdk4 (34 kDa) expression in BV2 microglia treated with HDACi or LPS or both. The data are normalized with β-actin (42 kDa) immunoreactivity (B). Significant differences between groups (control *vs* LPS, LPS *vs* HDACi and LPS *vs* HDACi +LPS) are indicated by *p<0.05, **p<0.01 (n = 3).

The effect of HDACi on the mRNA and protein expression levels of Cdk4 and LAT2 in control and LPS-treated microglia was analyzed. RT-PCR analysis showed that the mRNA expression of Cdk4 was significantly downregulated in HDACi and HDACi+LPS treated cells when compared to control (Fig. 10 A). However, LPS treatment alone induced the mRNA expression level of Cdk4 in microglial cells (Fig. 10 A). This was further confirmed by western blot analysis which showed a significant reduction of Cdk4 protein expression in HADCi and HDACi+LPS treated cells when compared to control and LPS treated cells (Fig. 10 B, C).

### Knockdown of Runx1t1 or HDACi Treatment Upregulates LAT2 which Reduces NO Production in BV2 Microglial Cells

**Figure 11:**
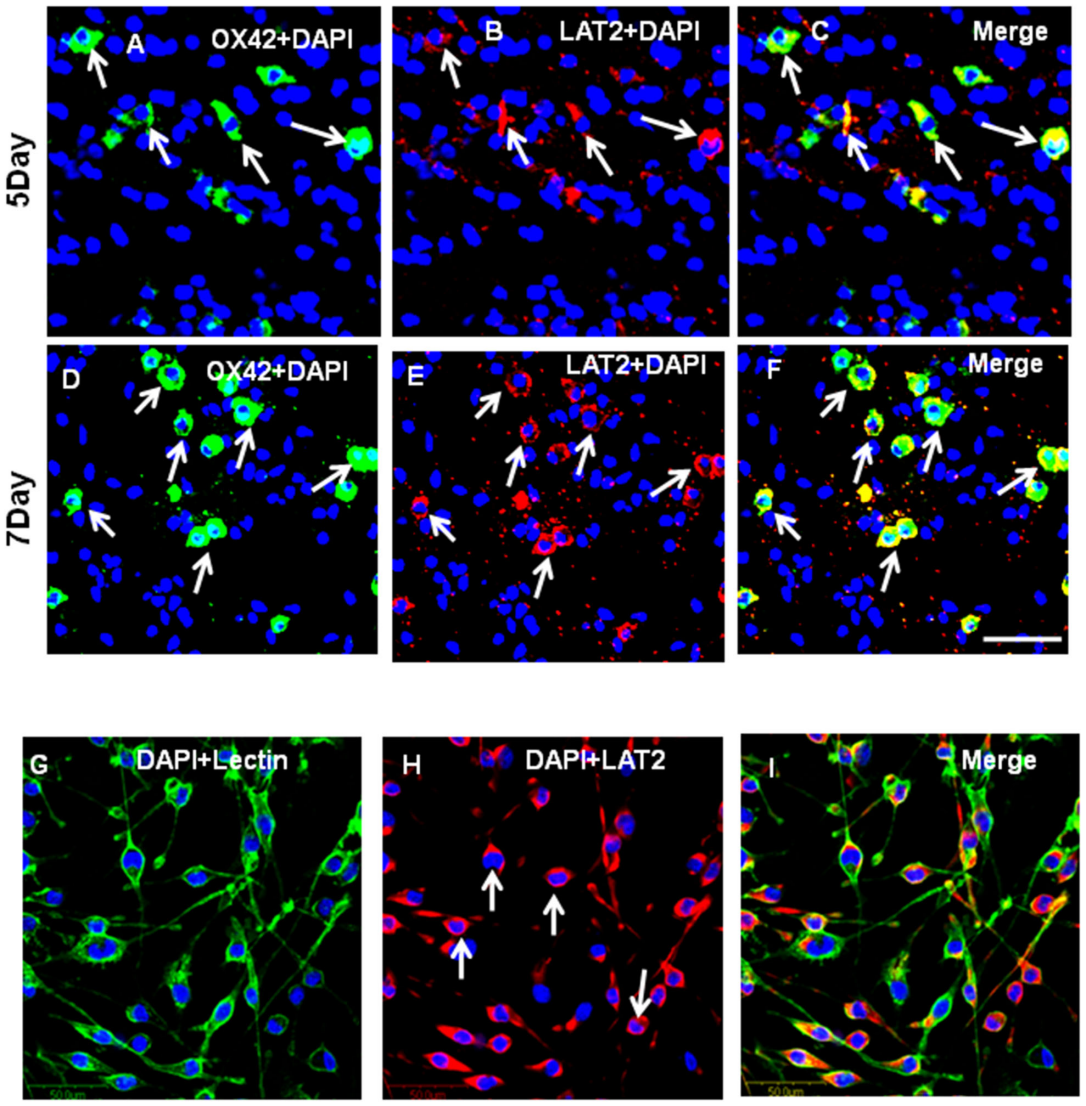
LAT2 expression in AMC and BV2 microglia. **A–F.** Confocal images showing the expression of LAT2 (B,E; red) in OX42-positive (A,D; green) microglia (C,F; yellow) from the corpus callosum of 5 &7days rat brains, respectively. **G–I.** Confocal images showing the expression of LAT2 (H; red) in BV2 microglial cells counter stained with lectin (G; green) and DAPI (blue).

**Figure 12:**
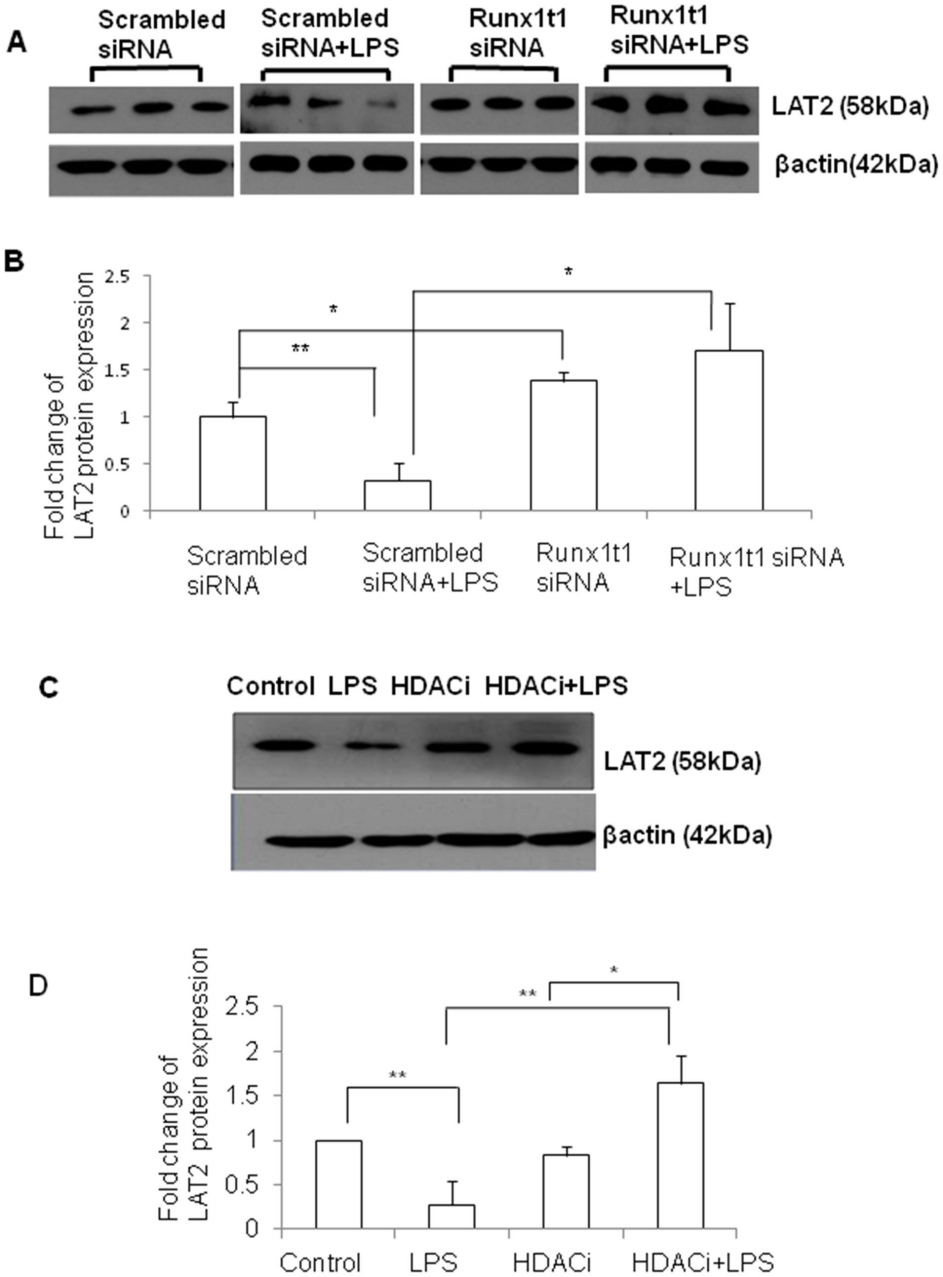
Expression of LAT2 protein inBV2 microglia transfected with Runx1t1 siRNA or treated with HDACi. **A–B.** Western blot analysis shows that expression level of LAT2 protein (58 kDa) is significantly downregulated in microglia treated with LPS, when compared to that transfected with scrambled siRNA. On the other hand, knockdown of Runx1t1 significantly upregulated LAT2 protein expression in LPS-treated BV2 microglial cells. The data were normalized with β-actin (42 kDa). **C–D.** Western blot analysis further shows a significant upregulation of LAT2 protein expression in LPS-treated BV2 microglial cells when they were treated with HDACi. Significant differences between groups (scrambled siRNA *vs* scrambled siRNA +LPS, scrambled siRNA *vs* Runx1t1 siRNA, scrambled siRNA +LPS *vs* Runx1t1 siRNA+LPS, control *vs* LPS, LPS *vs* HDACi+LPS, HDACi *vs* HDACi+LPS) are indicated by*p<0.05, **p<0.01 (n = 3). Scale bars: 50 µm.

Immunofluorescence analysis showed that LAT2 was expressed in OX42-positive AMC found in the corpus callosum of 5–7 d old rat pups (Fig. 11, A–F) and BV2 microglial cells (Fig. 11 G–I). The protein expression of LAT2 was significantly upregulated in Runx1t1 knockdown BV2 microglial cells when compared to scrambled siRNA (Fig. 12A, B). We have analysed the protein expression level of LAT2 after HDACi treatment to check whether HDACi has a similar effect on LAT2 expression as Runx1t1 knockdown in BV2 microglial cells. Western blot analysis showed a significant upregulation of LAT2 protein expression in HDACi+LPS treated BV2 microglial cells when compared to control (Fig. 12 C, D), indicating that HDACi treatment derepresses LAT2 in LPS-activated microglial cells.

**Figure 13:**
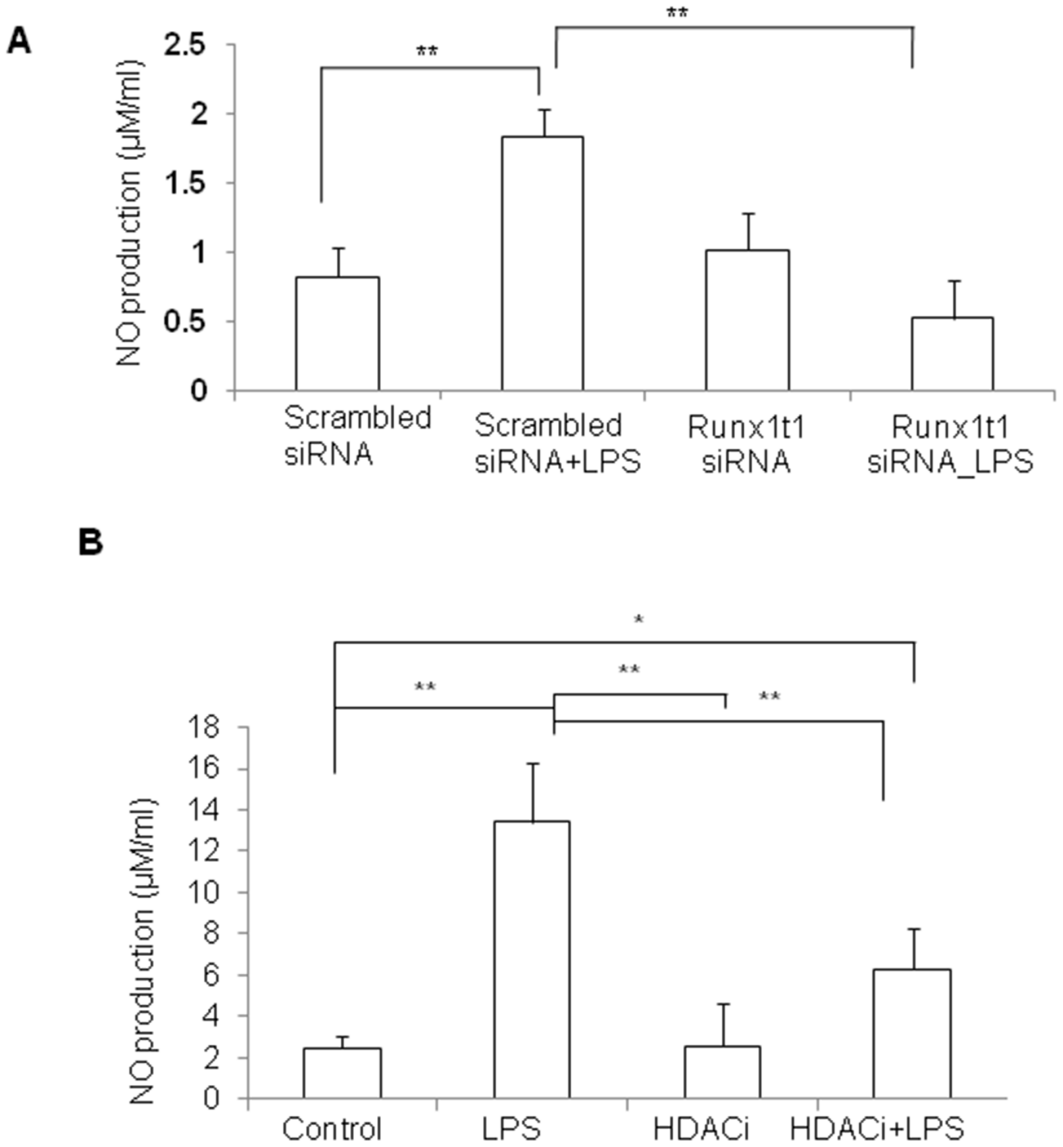
NO productionin BV2 microglia transfected with Runx1t1 siRNA or treated with HDACi. **A–B.** Nitric oxide (NO) assay analysis indicates that nitrite level is significantly increased in LPS-treated BV2 microglial cells and is brought down to near base level either by Runx1t1 siRNA knockdown(A) or by HDACi treatment (B). Significant differences between groups (scrambled siRNA*vs* scrambled siRNA +LPS, scrambled siRNA +LPS *vs* Runx1t1 siRNA+LPS, control *vs* LPS, control *vs* HDACi+LPS, LPS *vs* HDACi, LPS *vs* HDACi+LPS) are indicated by *p<0.05, **p<0.01 (n = 3).

It has been previously shown that enhanced expression of LAT2 contributes to a decreased production of NO [22]. We have measured NO production in activated BV2 microglia after knockdown of Runx1t1 or HDACi treatment (Fig. 13 A, B). NO assay analysis revealed that knockdown of Runx1t1 or HDACi treatment decreased the level of nitrite significantly as a consequence of induction of LAT2 in BV2 microglial cells (Fig. 13 A, B).

### ChIP Assay Reveals that HDACi Enhances the Binding of Runx1t1 to LAT2 Gene Promoter in Activated BV-2 Microglia

**Figure 14:**
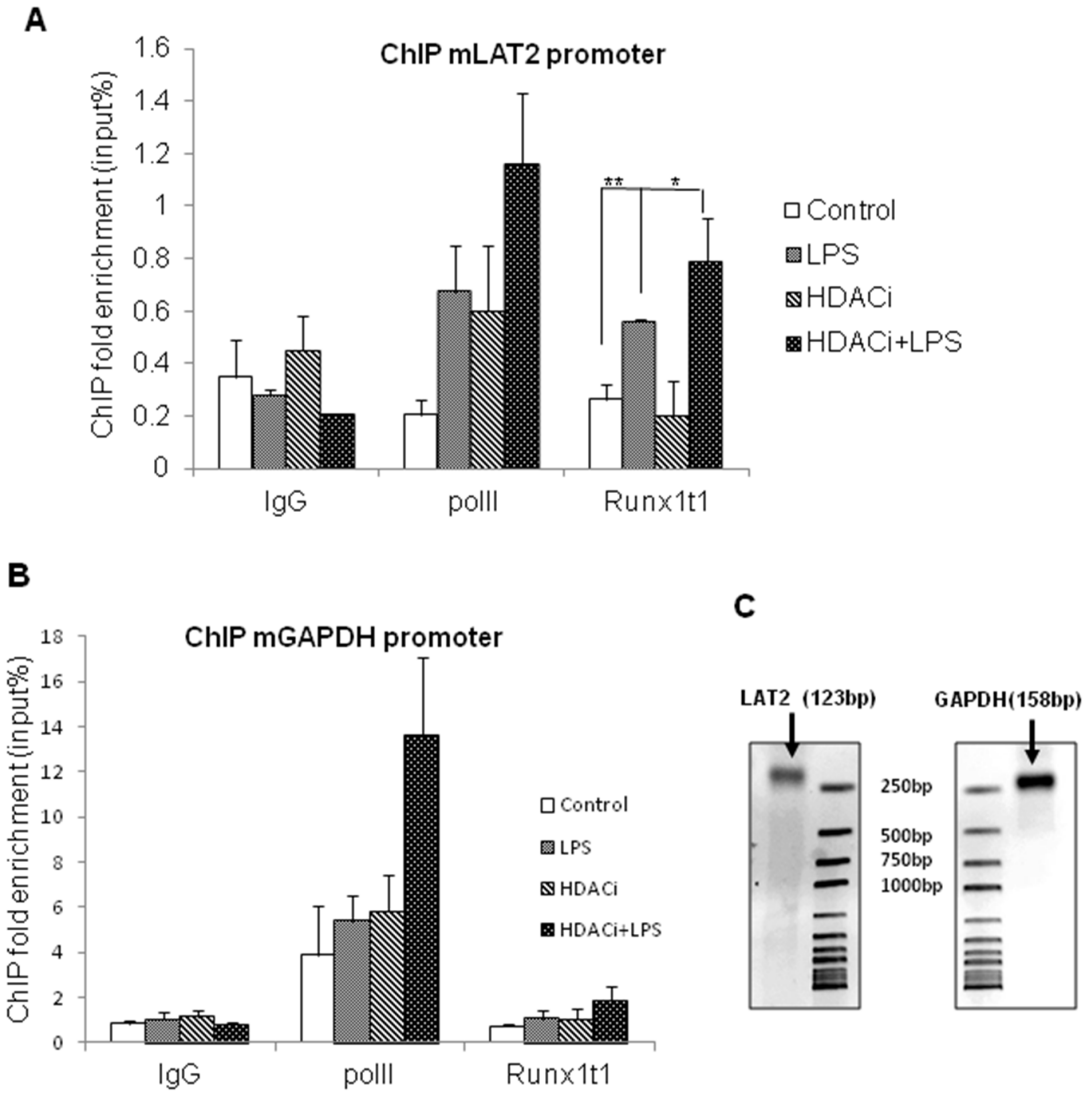
ChIP assay analysis showing the binding of Runx1t1 to LAT2 gene promoter. **A–C.** ChIP analysis revealed that binding of Runx1t1 to the promoter region of LAT2 is significantly increased in LPS-activated microglial cells treated with or without HDACi, when compared to control cells (Fig. 14 A,C). For all experiments, rabbit IgG was used as the negative control to exclude non-specific binding. Polymerase II binding at the GAPDH promoter was used as the positive control (Fig. 14, B,C). Significant differences between groups (control *vs* LPS, control *vs* HDACi+LPS, LPS *vs* HDACi+LPS) are indicated by *p<0.05, **p<0.01 (n = 3).

ChIP assay was carried out to investigate the interaction of Runx1t1 protein with LAT2 gene promoter using primers specific to LAT2 (Fig. 14A–C). ChIP analysis revealed that binding of Runx1t1 to the promoter region of LAT2 was significantly increased in LPS-activated microglial cells when compared to control cells (Fig. 14 A). Remarkably, HDACi treatment also increased the binding of Runx1t1 to the LAT2 promoter in LPS-activated microglial cells, compared to that of control (Fig. 14, A). Rabbit IgG was used as the negative control to exclude non-specific binding in all the experiments. Polymerase II binding at the GAPDH promoter was used as the positive control (Fig. 14 B, C).

## Discussion

In recent years, a large number of studies have attempted to understand the molecular mechanisms behind chronic microglial activation, as activated microglia have been shown to induce neurotoxicity through excessive release of inflammatory mediators and cytotoxic molecules [1], [4], [7], [34]. Identification and manipulation of transcription factors and secretory molecules that regulate microglial functions may help develop new strategies to limit this activation and subsequent neurotoxicity in neurodegenerative disease therapy. In the present study, Runx1t1, a transcription factor involved in the proliferation and differentiation of hematopoietic stem cells [12], [13] was found to be induced in activated microglia *in vitro* and in brains of TBI and AD rat models. The induction of Runx1t1 in activated microglia is noteworthy due to the fact that this gene was expressed normally in AMC which are highly motile and prolific, but not in adult quiescent microglia, and it plays a putative role in cell proliferation [35].

It has been well documented that microglial proliferation is one of the hallmarks in neuropathological conditions caused by infection, trauma or other neurological disorders [1], [2], [3]. Cell cycle progression is regulated by complex signaling pathways involving several molecules, including cyclin-dependent kinases (Cdk) [10], [11]. In the present study, knockdown of Runx1t1 in activated microglia downregulated the expression of cell-cycle related protein, Cdk4 and perturbed the proliferation of activated microglia indicating that Runx1t1 promotes microglial proliferation by regulating Cdk4 in neuropathological conditions.

Runx1t1 is known to recruit HDACs for transcriptional repression of target genes [13], [17], [18], [19], [36]. HDACs which catalyze histone deacetylation and chromatin condensation, are involved in controlling cell proliferation by regulating the cell cycle genes [20], [21], [37], [38]. HDAC-mediated chromatin remodelling upregulates the expression of Cdk4 which forms complex with cyclin D1 and phosphorylates retinoblastoma protein (pRb) which in turn regulates the G1/S transition in the cell cycle [20], [39], [40], [41], [42]. HDACi has been shown to induce deregulation of cell cycle genes, cyclin D1 and Cdk4 leading to hypophosphorylation of pRb [21], thereby resulting in cell cycle arrest in multiple myeloma cells. Further in the adult neural stem cells, HDACi treatment induced the transcriptional activity of Cdk inhibitors, such as p21 and p27 together with histone acetylation at proximal promoter regions, leading to cell cycle arrest [32].

In this study, HDACi (sodium butyrate) was used to block the availability of HDACs to Runx1t1 for regulating the transcription of its target genes in microglia. This led to a decrease in Cdk4 expression and mitotic index in activated microglial cells, thereby confirming the interaction between Runx1t1 and HDAC in regulating the proliferation of activated microglia. This finding is interesting as histone acetylation has previously been shown to reduce the proinflammatory response and activation of microglia [43]. Further, the present study also showed an increased histone acetylation in activated microglia following Runx1t1 knockdown as well as HDACi treatment. Together, these results strengthen the notion that Runx1t1 regulates the proliferation and proinflammatory response of activated microglia by interacting with HDACs.

In addition to increased proliferation, activated microglia secrete a plethora of proinflammatory cytokines and neurotoxic factors such as nitric oxide (NO) [7]. Recently, it has been reported that LAT2, a cationic amino acid transporter (y+ system) reduces NO production by depleting the availability of arginine to iNOS enzyme [22], [24]. The present study demonstrated that knockdown of Runx1t1 and HDACi treatment upregulated LAT2 gene expression, and suppressed NO production in activated microglia. It has also been demonstrated that LAT2 was repressed in activated microglial cells which showed upregulation of Runx1t1 and was derepressed in activated microglia upon Runx1t1 knockdown and HDACi treatment. These results clearly show that Runx1t1 represses LAT2 in activated microglia by recruiting HDAC for transcriptional repression. This was further confirmed by the promoter binding studies using ChIP assay which showed that the Runx1t1 protein binds to LAT2 gene promoter. Moreover, this binding was significantly increased in activated microglial cells cultured with or without HDACi, indicating that Runx1t1 binds to LAT2 promoter in microglia irrespective of the presence of HDAC, but it requires HDACs to transcriptionally repress LAT2, since LAT2 expression was upregulated in activated microglia after HDACi treatment. It appears that Runx1t1-induced transcriptional repression of LAT2 in activated microglia was abolished by the HDACi treatment as reported earlier [44]. Overall, this study provides convincing evidence that Runx1t1 contributes to microglia-mediated neuroinflammation by inducing microglia proliferation and their secretion of neurotoxic factor when activated *via* its interaction with HDACs and LAT2.

In conclusion, it has been demonstrated that Runx1t1 regulates Cdk4 expression possibly by recruiting HDACs, resulting in increased cell proliferation. In addition, Runx1t1 interacts with HDACs for repressing the expression of its target gene, LAT2, which normally reduces the production of NO in microglia. The repression of LAT2 expression results in increased release of NO which may contribute to neurotoxicity. It is suggested that the knockdown of Runx1t1 or the inhibition of HDACs could revert the epigenetic changes in target genes such as LAT2, thereby inhibiting microglial activation in neuropathological conditions.
